# Mechanisms of sharp wave-ripple generation and autonomous replay in a hippocampal network model

**DOI:** 10.1186/1471-2202-14-S1-O13

**Published:** 2013-07-08

**Authors:** Szabolcs Káli, Eszter Vértes, Dávid G Nagy, Tamás F Freund, Attila I Gulyás

**Affiliations:** 1 Institute of Experimental Medicine, Hungarian Academy of Sciences, Budapest, Hungary; 2 Pázmány Péter Catholic University, Faculty of Information Technology, Budapest, Hungary; 3 École Polytechnique Fédérale de Lausanne, Switzerland

## 

Several distinct patterns of population activity can be recorded in the hippocampus in vivo. These neural activity patterns depend on the behavioral state of the animal, and include theta-modulated gamma oscillations as well as low-level irregular activity with periodically occurring large-amplitude sharp wave-ripple (SWR) events. During SWRs, neuronal populations in the hippocampus have been found to "replay", on a faster time scale, activity recorded during theta-gamma activity in the exploring animal. Such replay may be important for the establishment, maintenance and consolidation of long-term memory. Our aim was to develop a mechanistic understanding of cellular and network mechanisms underlying the generation of SWRs in general, and spatio-temporal sequence replay during SWRs in particular, based on in vitro and in vivo experimental observations.

A recently developed hippocampal slice preparation, in which SWRs arise spontaneously, has allowed the collection of a large and diverse set of data regarding the properties of SWRs, as well as the characterization of several cell types and synapses which are critical in their generation. Based on these data, combined with phase-plane analysis of the network dynamics, we developed a large-scale network model of the hippocampal CA3 region. We found that our model based on measured cellular and synaptic parameters could faithfully reproduce the experimentally observed SWR activity as long as we included an appropriate slow feedback mechanism which was responsible for the termination of SWR bursts. Our model allowed us to rule out several potential candidates (such as neuronal adaptation) for the slow feedback process, suggesting that either slowly activating interneuronal feedback or short-term synaptic plasticity of connections within CA3 might terminate SWRs. Statistical analysis and fitting of the inter-event interval distribution of SWRs in vitro suggested that, following an initial 'refractory period' after each SWR, the next SWR is initiated stochastically, requiring the simultaneous activation of a threshold number of pyramidal cells. When we implemented the changes in cellular and synaptic properties measured in the slice following the activation of cholinergic receptors, our model replicated the experimentally observed transition from SWR activity to gamma oscillations. Finally, applying a spike-timing-dependent plasticity rule to the recurrent excitatory weights during simulated exploration, the emerging weight structure led to the spontaneous replay of sequences of place cell representations during simulated SWRs. We also found that using structured rather than random weights substantially altered the global network dynamics, resulting in a sparse participation of pyramidal neurons in individual SWR events, and allowing our model to match more closely the corresponding experimental observations.

